# The general attributes and competence for nurses in a single responder unit: A modified Delphi study

**DOI:** 10.1186/s12873-023-00868-w

**Published:** 2023-08-21

**Authors:** Viivi Tikkanen, Marija Arsic, Maria Henricson

**Affiliations:** 1 Faculty of Caring Science, Work Life and Social Welfare, University of Borås, Borås, Sweden; 2 School of Health, Care and Social Welfare, Mälardalens University, Eskilstuna, Sweden; 3 Falck Ambulance Stockholm, Stockholm, Sweden; 4 Emergency Department, North Älvsborg County Hospital, Trollhättan, Sweden

**Keywords:** Ambulance, Communication, Competence, Nurse, Patient safety, Single responder unit

## Abstract

**Aim:**

The aim of this study was to describe the general attributes and competence that nurses in the ambulance’s single responder units are considered to need.

**Background:**

The development of ambulance care has led to an increased need for new units and working methods. Single responder unit is a single crewed unit that often uses for the patient assessments, to refer patients to the right level of care and to release regular ambulances. There is a lack of description of the needed competence for the nurses within single responder unit.

**Methods:**

Modified Delphi with three rounds was used. The first round was conducted with focus group interviews and analysed with content analysis. Five competence categories and 19 subcategories were identified. The second and third rounds were conducted through surveys using a 4-point Likert scale and analysed with descriptive statistics.

**Results:**

The ability to communicate with other healthcare providers to achieve one’s goal, the ability to create a good encounter alone and to have appropriate professional experience were identified as the most important general attributes and competencies.

**Conclusions:**

A central competence in prehospital emergency care is the ability to independently assess and treat patients with varying care needs in complex environments. To be able to work in SRU requires good communication and collaborations skills with other healthcare providers but it is also a prerequisite for creating a good patient relationship. Work experience of taking care of varying patients and situations is also needed in SRU.

**Supplementary Information:**

The online version contains supplementary material available at 10.1186/s12873-023-00868-w.

## Background

Ambulance care has developed from an activity that once focused on the transport function to complex high-tech prehospital emergency healthcare in just a few decades [1–4]. At the same time, ambulance response times have increased [5–7] due to the increased frequency of ambulance missions and more advanced assessments and interventions [8]. For this reason, increased ambulance availability and reduced waiting times have become important goals in prehospital emergency care [6, 8]. Reaching these goals has increased the need for more resources in prehospital emergency care [3]. New kinds of life-saving units [9] are needed because of the longer ambulance response times [6]. The single-responder unit (SRU) is a single-crewed acute ambulance care unit charged with patient assessment, making decisions about the appropriate level of care, and initiating urgent and life-saving interventions [5, 10–12]. Its’ function and purpose vary in Sweden and internationally [5], as the unit is used both to improve access to ambulance care in rural areas [5, 13] and to relieve primary care [14]. In Sweden the SRU is staffed by a registered nurse [5] with or without a specialist education and equipped like a traditional ambulance but without a stretcher [5, 12]. Sweden is one of the countries where registered nurse with 3-year bachelor’s degree from university is considered as a most suitable competence for working in the ambulance care settings. The one-year specialist programme within ambulance care for RNs in Sweden is at the master level [15]. Results for time and quality perspectives for Sweden’s first SRU, established in 2013, were positive. Average response times decreased from 26 min to 13 min (49% reduction), while the percentage increase in patients reached by SRUs in a 20-minute time frame at acute alarm clearly increased from 21.9 to 88.1% [5]. Organising new ambulance care protocols also raises criticism. An increased risk of jeopardising patient safety and person-centredness can be linked to patient assessments and referrals conducted by ambulance nurses. The use of various resources that initiate acute manoeuvres on the patient before the arrival of the ambulance can also increase this risk [7]. Dealing with different patients, assessing their care needs, and providing care alone is a further challenge [16]; therefore, close collaboration is needed between SRUs and other healthcare providers [13]. From a work-environment and safety perspective, working alone within SRUs can increase staff risks regarding threats and violence [12, 15]. These issues point to the importance of SRU staff competence. Competence can be seen as an individual’s ability to perform a job based on theoretical knowledge, practical skills and reflective ability [17]. Formal competence is generated through education, while real competence arises from professional life or everyday situations [18]. The professional competence of healthcare staff is a prerequisite for good and safe patient care [19], while a lack of competence raises the risk of healthcare injuries [20, 21]. Poor education, clinical judgment and decision-making skills are the greatest risks to patient safety in ambulance care [22]. The constant development of prehospital emergency care has led to a lack of clearly defined competence requirements within ambulance care [17]. The Swedish Nurses’ Association [23], Wihlborg et al. [15] and Nilsson et al. [19] have identified competencies within the Swedish Emergency Medical Service (EMS); however, these are only presented in a context of traditional pair work. The ability to independently assess and treat patients with varying care needs in complex environments is a central competence in prehospital emergency care [7, 19]. Dixon et al. [24] reported that the use of SRUs can reduce both the need to transport patients to the hospital and the time they spend in the emergency department. Vicente et al. [12] indicated that patients appreciate the way nurses on SRUs work. Patient safety is perceived as high because the nurses take the time to determine different treatment options, obtain support from medical treatment guidelines and/or consult doctors to make informed decisions [12]. Brewster et al. [25] reported that high patient satisfaction may reflect a greater focus on a patient-centred perspective and more effective collaboration with other care providers among staff in SRUs. To promote SRU’s uniform and high-quality healthcare, a mapping of the needed competence is necessary. The purpose of this study was to identify the most important general attributes and competence that nurses in SRUs are considered to need.

## Methods

### Design

A modified three-round Delphi method [26] with a mixed-method design was used. The first round was conducted using a qualitative approach, in which data were collected through focus group interviews and analysed through an inductive content analysis. Five competence categories and 19 subcategories were identified (Table 1). The second and the third round was conducted using a quantitative approach. The data were collected through a digital survey. Total of 19 questions with 80 statements in survey based on the analysis of the interviews. A 4 -point Likert scale was used for the rating of the statements. Data were analysed through descriptive statistics, with mean value and standard deviation (SD). Data were automatically available in a survey tool Survey Monkey ®.

**Table 1:** Categories and subcategories

Category	Subcategory
Education and professional experience needed for duty	○ To have an appropriate formal competence ○ To have appropriate professional experience ○ To be able to increase one’s competence through internal education
Personal qualities needed for duty	○ To have personal suitability ○ To have social skills ○ To be able to create a good encounter alone ○ To be humble ○ To be able to take a leadership role
Ability to handle information effectively	○ To be able to communicate with different healthcare providers to reach one’s goal ○ To be able to document carefully ○ To master information and communication technology
Ability to work in a safe manner	○ To be able to work based on patient safety ○ To be able to work based on one’s own safety ○ To be able to handle vehicles and equipment in a safe manner
Developing one’s competence to perform optimal work	○ To have knowledge in improvement work and quality development ○ To be able to maintain one’s own competence ○ To be able to identify and present development needs and ideas ○ To obtain information and support for medical problems ○ To be able to develop oneself and create new skills

### Recruitment process and selection for the first round

Four regions in Sweden with SRU were selected and information letters were sent to operational EMS managers. The managers approved the study and provided contact information to potential experts, meaning ambulance nurses who met the following inclusion criteria: (1) formal education as a registered nurse with or without a specialist degree; (2) at least two years of work experience within SRU; and (3) willingness to share their opinions on the subject. Informants expressing interest in participating in the study gave informed consent via email and answered background questions (Table 2). Eighty-two potential experts were contacted. Twenty-two experts- from two regions showed interest in participating. After two dropouts, a total of 20 experts (7 women and 13 men, aged 35–63 years) participated in the interviews in the first round. One of the experts was a registered nurse and 19 experts had one or more specialist educations. The mean value for work experience for all the experts was 15.7 years in ambulance care and 4.8 years in SRUs.

**Table 2:** Participant demographics

Variable	Region A	Region B
Assignments/year		
Assignments in 2020	3846	3033
Assignments in 2021	3413	3041
Participants		
All, n (%)	11 (55)	9 (45)
Women, n (%)	2 (10)	5 (25)
Men, n (%)	9 (45)	4 (20)
Education		
Registered nurse, n (%)	1 (9,1)	0
One specialist nurse education, n (%)	6 (54,5)	8 (88,9)
Two or more specialist nurse educations, n (%)	4 (36,4)	1 (11,1)
Years worked within ambulance		
< 10 years, n (mean value)	0	2 (9,0)
10–20 years, n (mean value)	8 (14,8)	7 (14,6)
> 20 years, n (mean value)	3 (22,3)	0
Years worked within SRUs		
2–3 years, n (mean value)	8 (2,0)	1 (3,0)
4–5 years, n (mean value)	1 (4,0)	2 (5,0)
6–9 years, n (mean value)	2 (7,5)	6 (7,8)

### Data collection for the first round

Five semi-structured focus group interviews were conducted in March–April 2022 [27, 28], via the Zoom® digital video meeting tool. The roles of moderator and bystander were chosen between the authors (V.T and M. A) before each interview [27]. The interviews, with a support of a PowerPoint presentation, lasted average 70 min (Table 3) and were audio recorded with an external dictaphone for later verbatim transcription. The interview questions are presented in Appendix 1.

**Table 3:** Overview of the participants of each interview and the length of the interviews

Group interview	Length	Number of participants Region A	Number of participants Region B	Total number of participants
1	96 min	3	0	3
2	67 min	4	0	4
3	75 min	0	4	4
4	56 min	3	2	5
5	58 min	1	3	4
Total quantity	5 h 25 min	11	9	20

### Data analysis for the first round

The focus group interviews were analysed according to Graneheim and Lundman [29]. The analysis process, using an inductive approach, was started in connection with the transcription by delving into and increasing the understanding of the data material. Initially, all transcripts were read repeatedly. In total, 357 meaning units were identified. The meaning units were summarised into condensed meaning units. A code was then added to the condensed meaning units. Differences and similarities between the codes were identified, and codes with similar content formed 19 subcategories. Further subcategories were then formed according to the code similarities, and categories were later formed according to the differences and similarities between the subcategories [29] (Table 4). The analysis was carried out in parallel with different parts of the data to simultaneously create an overall picture and correctly interpret the details.

**Table 4:** Example of content analysis for two categories

Sentence unit	Condensed sentence unit	Code	Subcategory	Category
*“But I think the personal suitability is what should weigh the most.“* (Informant #12)	Personal suitability weighs the most heavily.	Personal suitability.	Having personal suitability.	Personal qualities needed for duty.
*“You are alone, and words stand against words. You have to be extremely thorough so that you really get everything, how you think and how the patient has perceived the whole thing.“* (Informant #6)	Word for word, accurate documentation is essential so that both the patient’s own words and the patient’s opinion are included.	Accurate documentation.	Being able to document carefully.	Ability to handle information effectively.

### Recruitment process and selection for second round

Two weeks after the last focus group interview, a link to a digital survey on the SurveyMonkey® was sent via email to the 20 experts who participated in the interviews. The email contained compiled feedback from the interviews as well as information about the time estimated for completion of the survey (10–15 min). The experts interested in participating in the second round gave their informed consent by answering the survey questions. After three dropouts, 17 experts participated in the second round.

### Data collection for the second round

The second round was conducted using a quantitative approach, and data were collected through a survey in SurveyMonkey ®. To develop a survey based on the experts’ thoughts and claims, content was selected based on 80 statements under 19 subcategories that the experts had expressed during the focus group interviews. One subcategory could contain one till seven statements, for example:

To be able to handle vehicles and equipment in a safe manner (subcategory).

- *Being able to multitask and at the same time take care of information management, communication, and the driving of vehicle* (One statement, based on interviews, used in a survey for the round two and three.) and

Ability to develop oneself and create new skills (subcategory).

- *In-depth knowledge in assessment, geriatrics and psychiatry is needed to work on SRU*
- *In-depth knowledge in assessment and geriatrics is needed to work on SRU*
- *In-depth knowledge in assessment and psychiatry is needed to work on SRU*
- *In-depth knowledge of geriatrics and psychiatry is needed to work on SRU*
- *In-depth knowledge of assessment is needed to work on SRU*
- *In-depth knowledge of geriatrics is needed to work on SRU*
- *In-depth knowledge of psychiatry is needed to work on SRU*
  (Seven statements, based on interviews, used in a survey for the round two and three.)

Feedback from round one to experts was given in two ways; a compiled information about the results based on content analysis was sent via email and experts got the feedback in form of survey where competencies highlighted in the first round were used to design the survey [28]. Six ambulance nurses’ pilot-tested the survey. Their responses were not included in the study. The experts were asked to rate a total of 19 subcategories with 80 statements using a 4-point Likert scale [30]. Rating was as followed: 1 = No importance, 2 = Of average importance, 3 = Great importance and 4 = Very great importance. The experts could navigate among the questions and go back and forth to correct their answers in the survey. Four reminders were sent to the experts via email during the data collection period.

### Data analysis for the second round

Descriptive statistics, with mean value and standard deviation (SD), were used [28]. After the second round consensus was reached for 15 of the 19 subcategories, and for 37 of the 80 statements. Consensus within each subcategory was achieved when ≥ 70% of the experts rated at least one of the statements exactly same under each subcategory by choosing one of the rating alternatives under each subcategory.

For example, subcategory *To be able to master information and communication technology* achieved consensus when 82,35% of experts rated statement *Mastering effective verbal communication using radio communication system and telephone* as Very great importance (4 in the Likert scale) resulting a mean value 3.82 (please see Table 5). Every Likert scale rate between one and four was analysed separately to be able to form a detailed result.

**Table 5:** Expert estimation of the needed competence that achieved consensus

Subcategory	Statement	Percent	Mean value	SD
To have an appropriate formal competence	District nurse*	73.33*	2.87*	0.50*
To have appropriate professional experience	More than 5 years of professional experience in pre-hospital emergency care	76.47	3.65	0.76
	Broad professional experience is required; that you met different patients in varying situations	94.12	3.88	0.47
	Extensive experience in situations where intensive work is required; for example, cardiac arrest and care of critically ill patients	76.47	3.76	0.42
To be able to increase one’s competence through internal education	If necessary, could be able to settle into several work roles at the same time	88.24	3.82	0.51
To have personal suitability	Personal suitability is not as important a factor as formal competence and previous professional experience	82.35	1.18	0.38
	Formal competence, previous professional experience and personal suitability are all equally important factors	70.59	3.41	1.03
To have social skills	That the nurse has patience and can maintain a calm and safe approach to patients and relatives	82.35	3.82	0.38
	That the nurse can express herself verbally in a clear way, can prioritise the flow of information from different people and construct a mutual dialogue with the patient and next of kin	82.35	3.82	0.38
	That the nurse has the ability to face mistrust on the part of the patient and relatives and transform it into trust and confidence and not allow herself to be provoked in the meeting with the patient and relatives	82.35	3.82	0.38
	That the nurse has a clear patient perspective in her humble treatment of patients and relatives	76.47	3.65	0.68
To be able to create a good encounter alone	In the encounter within the SRU, you need to show commitment to the patient and relatives	88.24	3.88	0.32
	In the encounter within the SRU, you need to have a broad approach	88.24	3.88	0.32
	In the encounter within the SRU, you need to be able to read people and situations	94.12	3.94	0.24
	In the encounter within the SRU, you need to be able to convey calm and take time to meet the needs of the patient and next of kin	88.24	3.88	0.32
To be humble	Humility towards colleagues is important, they should not get the feeling that I, a nurse in the SRU, am in charge	76.47	3.71	0.57
	Humility in the face of the fact that I too may need help from others is important	76.47	3.76	0.42
	In the SRU, it is important to show respect and humility towards each other’s different tasks and roles	88.24	3.88	0.32
To be able to work based on patient safety	Careful medical history taking, examination and assessment	88.24	3.88	0.32
	To work calmly and structured and double check what has been done	76.47	3.76	0.42
To be able to work based on one’s own safety	Based on a high level of safety thinking, work proactively and avoid ending up in risky situations	88.24	3.88	0.32
	To prevent threatening situations through one’s calm and humble behaviour and approach	76.47	3.65	0.68
	Ability to establish contact with the alarm centre during risky assignments	76.47	3.76	0.42
To be able to handle vehicles and equipment in a safe manner	To work proactively and prepare for assignments by handling information management and communication before driving vehicles	88.24	3.88	0.32
	To work from a safety perspective and if necessary for information management and communication while driving stop for a while	76.47	3.76	0.42
To be able to communicate with different healthcare providers to reach one’s goal	Nurses in the SRU have the ability to create contact routes with different healthcare providers	100	4.00	0.00
	The nurse in the SRU needs to present her case to other healthcare providers in a way that will lead to the desired goal	82.35	3.76	0.55
	Nurses in the SRU have the ability to utilise different resources and other people’s skills	88.24	3.88	0.32
To be able to document carefully	What has been said needs to be achieved through clear and accurate documentation	82.35	3.82	0.38
	Whether or not you agree with the patient and next of kin needs to be carefully documented	76.47	3.76	0.42
	That the patient refuses to go in despite our and the physician’s recommendation needs to be carefully documented	88.24	3.88	0.32
To be able to master information and communication technology	Mastering effective verbal communication using radio communication system and telephone	82.35	3.82	0.38
To be able to maintain one’s own competence	To share experiences and knowledge between ambulance colleagues	70.59	3.65	0.59
	To follow up the patient through the medical record after assignment	82.35	3.82	0.38
To be able to identify and present development needs and ideas	SRU staff need education days that are specifically targeted and adapted to SRU operations and work	70.59	3.53	0.78
To obtain information and support for medical problems	Leaning towards medical treatment guidelines	70.59	3.71	0.46
	To retrieve information via internet pages, e.g., Swedish Poisons Information Centre	70.59	3.59	0.69
	To discuss with the patient and next of kin	76.47	3.65	0.68

*Results from the third round

### Recruitment process and selection for the third round

**Fig. 1:**
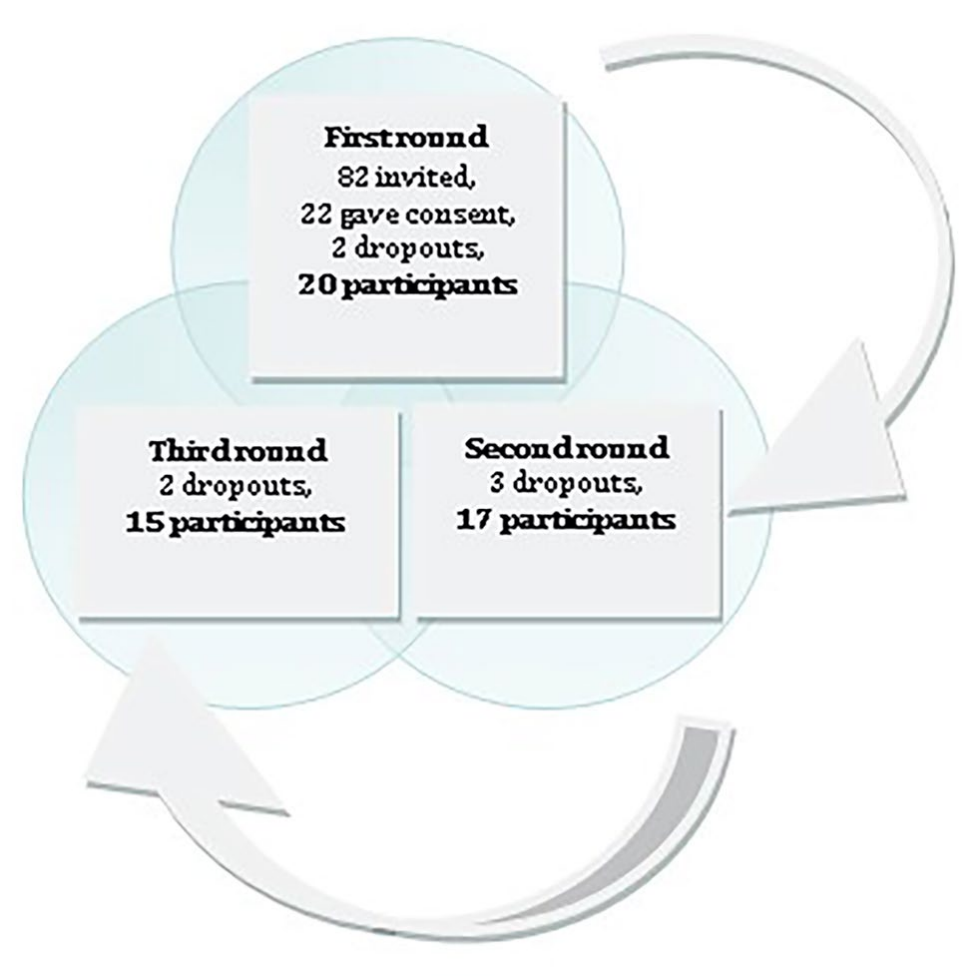
Number of participants per round Each block presents the number of experts that participated in the study as well as those who decided not to participate further in the second and third rounds

The third round was conducted with a quantitative approach, with data collected through a survey in SurveyMonkey ®. Before the third round, an email with the same structure as before the second round was sent out to all 20 experts who had participated the first round. Participation in the second round was completely anonymous; therefore, it was not clear who had completed the survey. Two experts chose not to participate the third round, resulting in 15 of the 20 original experts completing the entire study (Fig. 1).

### Data collection for the third round

**Fig. 2:**
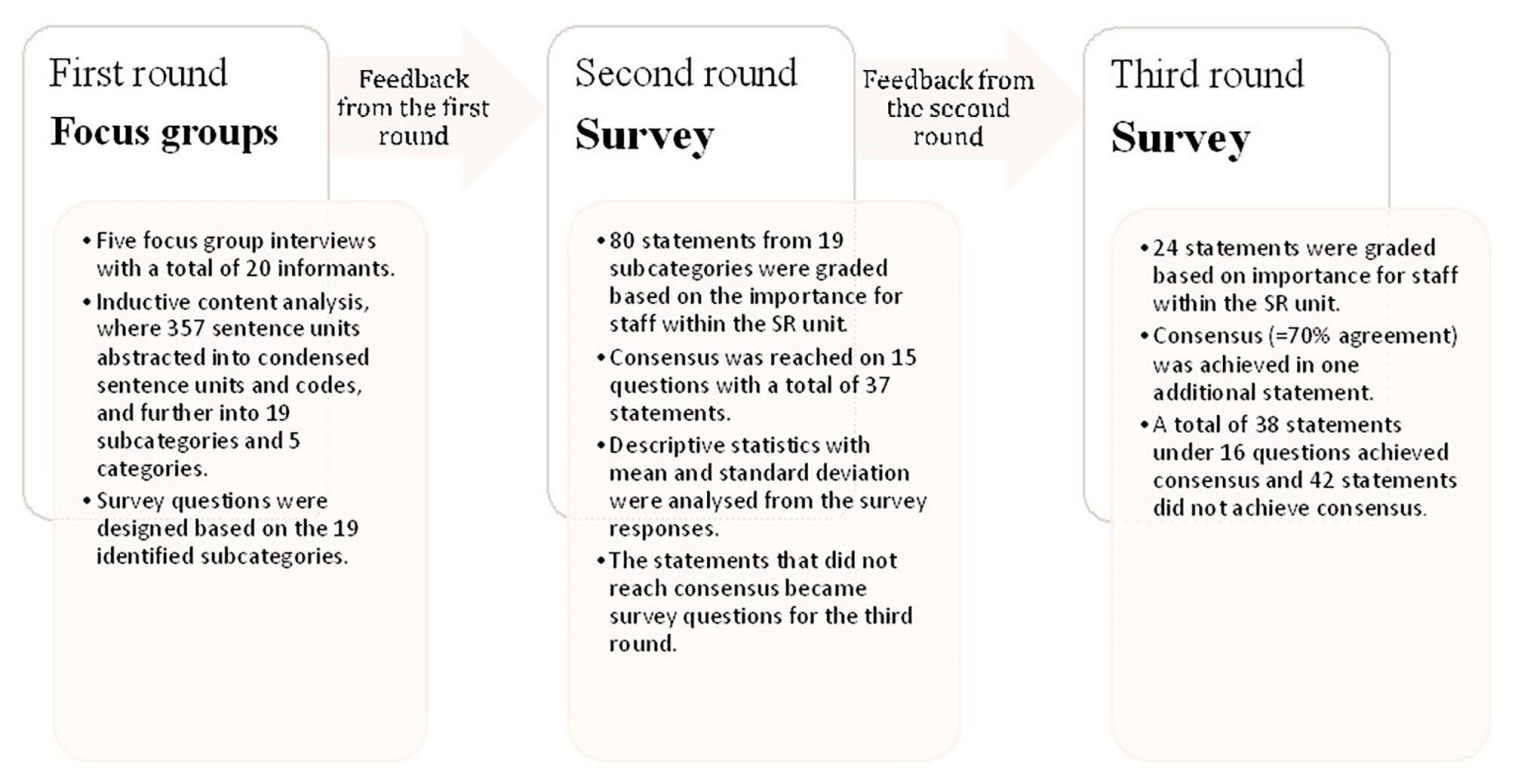
Data collection over three rounds according to modified Delphi method The figure is showing how data was collected in this study over here rounds according to modified Delphi method

Feedback after round two was sent as a compiled information about the results under the second round in group level. Each expert had also an opportunity to go in the survey in SurveyMonkey ® and see their own individual results for the round two, to be able to compare them with the groups’ results. Four questions, with a total of 24 statements that had not achieved consensus in any of the statements in the second round, were sent again to the experts. As in the second round, a 4-point Likert scale was used. In the third round, four reminders were sent out. The data collection process is presented in Fig. 2.

### Data analysis for the third round

Statistical data, which were automatically available via SurveyMonkey®, revealed after the third round that consensus (at least 70% agreement to one or more statements per question) was reached on another statement under one of the questions. Finally, consensus was reached for 16 of 19 questions under one or more statements. Descriptive statistics, with mean and standard deviation (SD), were used [28].

### Ethical considerations

The experts were fully informed, both verbally and in writing, about the aim of the study and the voluntary nature of participation, as well as the possibility of withdrawing. Experts provided their informed consent via email prior to data collection and the experts were guaranteed total confidentiality in accordance with the Declaration of Helsinki [31]. The experts were not close colleagues and not in a state of dependency to the authors. In accordance with the Swedish law regarding the ethical review of research on human beings, approval from Research Ethics Committees for interviewing staff in their profession was not needed (SFS 2003: 460; SFS 2019:1144) [32, 33]. Therefore, the institutional review board at University of Borås, Faculty of Caring science, Work Life and Social Welfare was consulted and approved the study.

## Results

Consensus (≥ 70% agreement) was achieved in 15 of 19 subcategories and in 37 of the 80 statements after the second round. Table 5 presents the mean value from a 4-point Likert scale and the SD of each statement. After the third round, consensus was reached for one more statement under one question. Overall, consensus was reached on 38 of the 80 statements asked, under 16 of the 19 subcategories. The strongest consensus, with 100% agreement, was reached for the opinion regarding the nurse’s ability to create contact routes with other healthcare providers. A consensus with 94% agreement was achieved regarding broad professional experience and the ability to read people and situations. A high consensus of 88% was achieved in ten statements. All these competences were considered as being of very great importance. Skills which were considered important for ambulance nurses in SRU in the encounter with patients were to show commitment, convey calmness, use a broad approach towards the patient’s situation and broad problem-solving ability. The ability to master several work roles at the same time, use other healthcare providers and their competence, but also to show respect and humility towards other healthcare providers were competencies that ambulance nurses in SRU need to have. Careful medical history taking, examination and assessment and proactive working methods, were also considered as important competencies.

After the third round, 73% of the experts reached a consensus that the district nursing education has a great importance for the work within SRU. No consensus was reached regarding the importance of being a RN or having a specialist degree in ambulance care or anaesthesia. Most experts considered that more than five years’ experience in ambulance healthcare and long experience in demanding situations were particularly important. The experts further considered that formal competence, previous professional experience, and personal suitability were all equally important factors for working in SRU. The greatest variation occurred in the experts’ answers about the ability to develop oneself and create new skills, internal education and improvement work and quality development.

## Discussion

**Fig. 3:**
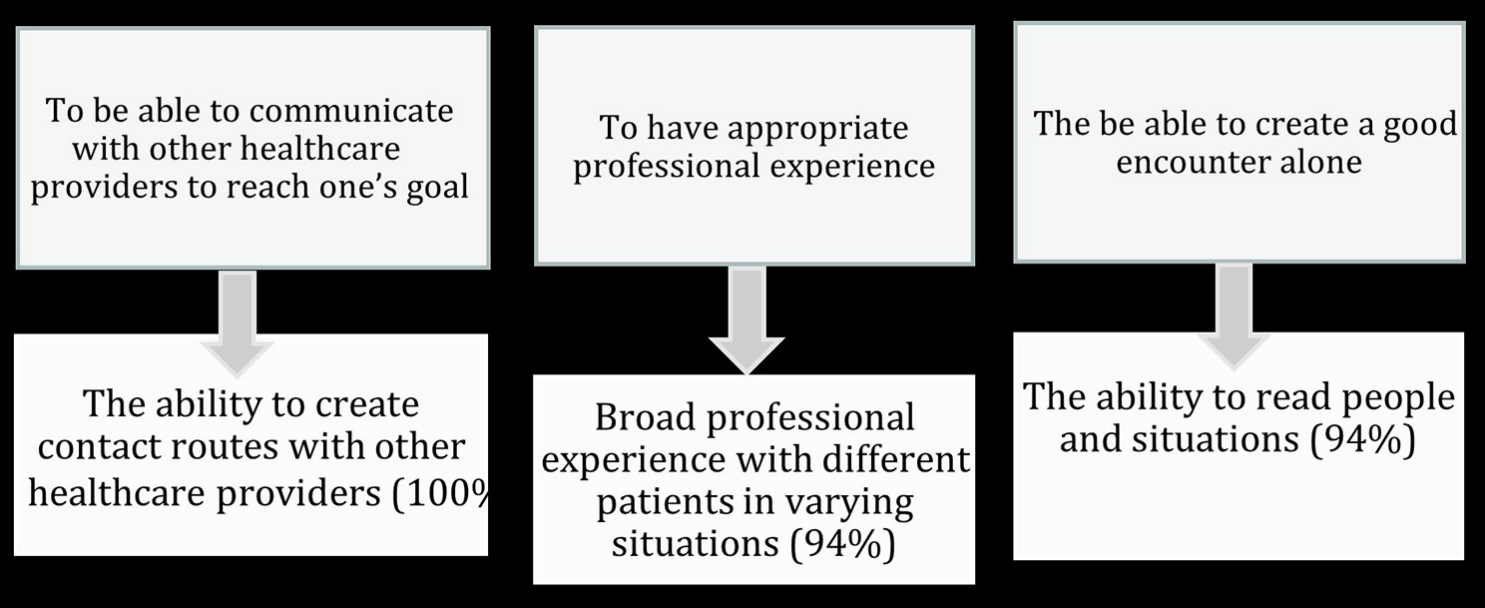
The main findings The figure presents the main findings of the present study. These three competencies (subcategories) achieved the highest rate and consensus; to be able to communicate with other healthcare providers to reach one’s goal with the statement the ability to create contact routes with other healthcare providers which was seen of very high importance of all experts (100%), to have appropriate professional experience with statement broad professional experience with different patients in varying situations and to be able to create a good encounter alone with statement the ability to read people and situations were both seen as of very high importance of 94% of experts

The purpose of this study was to identify the most important general attributes and competence that nurses in SRUs are considered to need. Three of the competencies achieved a remarkable consensus with very high importance. Competencies and their meaning are illustrated in Fig. 3 and are the main results of the study.

Intensive cooperation and communication between other healthcare providers were described as central to the everyday work within SRU in this study. A good collaboration between other healthcare providers is linked to the patients’ improved care results and care experience [34]. The participation of patients and next of kin in decision-making was a high priority. This result can be linked to a person-centred perspective that focuses on promoting patient participation and involvement in their own care [35]. The ability to create contact routes and communicate and collaborate with other healthcare providers, which considered as needed competence for the SRU nurses, represents skills that are required to work independently from a person-centred and proactive perspective [3, 14, 36, 37]. The ability to read the existing situation and the people involved in it was considered important from both a person-centred perspective and a safety aspect. Working within SRU often offered more room to create a good relationship with a patient, but sometimes the absence of a colleague increased the feeling of vulnerability. According to Adio et al. [38], a calm encounter without haste provides a basis for good social interaction within SRUs. As in this study, previous research has identified that broad professional experience and social competence are necessary competencies within the SRU [11, 14, 37]. Broad experience was considered to mean several patient contacts in different situations in a prehospital context. On the one hand, working with critically ill patients were considered valuable experience; on the other hand, experience in primary care was emphasised. Furthermore, education as a district nurse was considered the most appropriate formal competence within the SRU. In Sweden, district nurses are specialist nurses [39]. The programme for specialization in district nursing is 1.25 years, and before participating the programme students need to have a Bachelor of Science degree in nursing [40]. District nurses’ responsibilities include preventing illness in the population and planning, providing, and evaluating care at primary health care centres and in-home health care. District nurses spend a large proportion of time caring for older adults [39].

This result reflects changes in the patient demographics and working methods of ambulance healthcare, as well as the entire healthcare paradigm shift that now replaces the traditional role characterised by blue lights and sirens with the SRUs’ “softer role” [14, 36, 41]. Eaton et al. [42] point out that only limited information is available regarding the competencies needed to work within SRUs. SRU’s function which rather aims at patient assessment more than treatment means, from an international perspective, that the ambulance nurse’s needs, in addition advanced clinical skills, be adept at assessing and managing chronic diseases, mental illness and mapping social needs [42–44]. Competencies that achieved consensus and are considered as important ones in this study are similar with the staff in the similar units than SRU needs to have in other countries, for example in United Kingdom [42], in Finland [14] and in Australia [44]. A standardisation of the function and competence requirements of SRUs in Sweden is needed. This need is also identified internationally in numerous previous studies [3, 13, 25, 36]. Uniform national education for ambulance nurses in SRU would ensure reliable and uniform care [38].

### Methodological considerations

The chosen method allowed the aim of the study to be addressed, and a relevant choice of method is a prerequisite for the validity of a study [28, 29]. Ambulance nurses who met the inclusion criteria were seen as experts which can be seen as a weakness because the experience requirement was not more than two years’ work experience in SRU. Nevertheless, had experts worked many years in the prehospital emergency care. The variation in focus groups may have caused an uneven group dynamic and group bias [26]. The principle of anonymity, which is central to a Delphi method [26], could not be respected during data collection for the first round because, during the focus group interviews, the experts could see each other. However, participation in the second and third rounds was anonymous. One methodological weakness was that because of the content analysis related to first round it was not possible to send the individual feedback to experts after the first round, which is desirable according to main principles of a Delphi method [26]. The anonymity caused that it was unclear if the dropouts between round two and three were same experts or if the ones dropping out in round two decide to answer the survey in round three. The validity of the study was strengthened by pilot testing of the survey. The automatic process of quantitative data analysis through SurveyMonkey® can be considered to increase the study’s replicability [28]. The feasibility of this study is reinforced by the fact that different parts have been clearly reported [29]. Since the opinions of experts constitute the lowest level of the hierarchy of evidence [45], general competence requirements for SRU nurses cannot be definitively established by this study. Nevertheless, its results provide valuable information about the real competencies that the work within SRU requires.

### Implications for practice

Nurses working within SRU needs several competencies in addition to the competencies associated with their traditional roles with regular ambulances. The care that SRUs should be able to offer has elements from primary care, geriatrics, and psychiatry, in addition to emergency care. Collaboration and good communication between SRUs and other healthcare providers increase the opportunities for person-centred care, and a proactive approach within SRUs is necessary. The work within SRUs is carried out alone; therefore, social competence plays a central role; and the ability to provide good care for patients and next of kin is a prerequisite for a caring relationships and correct assessment. The results of this study can be implemented in prehospital recruitment processes and used for education purposes. The district nurse was considered as the most suitable education for work within SRUs, even though the SRU is part of the EMS activities.

## Conclusion

A central competence in prehospital emergency care is the ability to independently assess and treat patients with varying care needs in complex environments. To be able to work in SRU requires good communication and collaborations skills with other healthcare providers but it is also a prerequisite for creating a good patient relationship. Work experience of taking care of varying patients and situations is also needed in SRU. Result from this study suggests that the district nurse could be the most suitable education for working within SRU, even though the SRU is part of the EMS. Further research on the most appropriate formal competence is still needed. The results of this study can be implemented in prehospital recruitment processes and used for education purposes.

### Electronic supplementary material

Below is the link to the electronic supplementary material.

Supplementary Material 1

## Data Availability

The qualitative datasets generated and analysed during the current study are not publicly available due to protect the anonymity and privacy of study participants. Participants did not consent to have their full transcripts or excerpts of transcripts made publically available but are available from the corresponding author on reasonable request. The quantitative data that support the findings of this study are available from SurveyMonkey® but restrictions apply to the availability of these data, which were used under license for the current study, and are not publicly available. Data are available from the authors upon reasonable request and with permission of SurveyMonkey®.

## Abbreviations

EMS: Emergency Medical Services
SRU: Single Responder Unit
