# Supplementary material for: The general attributes and competence for nurses in a single responder unit: A modified Delphi study
Source: BMC Emerg Med. 2023 Aug 21;23:93. doi: 10.1186/s12873-023-00868-w (PMC10441716; doi:10.1186/s12873-023-00868-w)
Supplement: Supplementary file 1 — Supplementary Material 1 [file 12873_2023_868_MOESM1_ESM.docx]

| 1. General questions | A. What education and professional experience should staff have to be able to work in an SRU unit?  B. How well does the formal education you have received via the institution of higher education correspond to the level of competence and tasks required to work in an SRU unit?  C. What skills are required to work in an SRU unit compared to working in a regular ambulance? |
| --- | --- |
| 1. Person-centred care | A. What competence is required to be able to deal with different patients alone within the SRU unit? |
| 1. Teamwork and collaboration | A. What skills are required for collaboration in the team?  B. What management skills do you think are required in the SRU staff? |
| 1. Evidence-based practice | A. If you encounter a medical problem, where and how do you look for the facts and knowledge necessary to solve the problem?  B. How can you maintain and update your skills?  C. Is there a need for internal education and, if so, what kind of? |
| 1. Quality improvement | A. What roles do SRU staff have in improvement work and quality development of their own operations? |
| 1. Safety | A. How do you approach patient safety given the risks associated with working alone?  B. How do you feel about your own safety considering the risks associated with working alone?  C. How do you feel about the risks associated with working alone based on the handling of vehicles and technical equipment? |
| 1. Informatics | A. What gives you the best conditions as a SRU staff for good communication and record keeping? |

**Appendix 1.** Interview guide.

Appendix 1 presents semi-structured interview guide which has been divided in seven categories inspired by Ellström's theory of professional competencies ^[18]^, Carlström’s and Fredén's ^[5]^ description of the SRU’s function and the nurses’ core competencies.
